# Connexin43 as a Tumor Suppressor: Proposed Connexin43 mRNA-circularRNAs-microRNAs Axis Towards Prevention and Early Detection in Breast Cancer

**DOI:** 10.3389/fmed.2019.00192

**Published:** 2019-08-28

**Authors:** Nataly Naser Al Deen, Mounir AbouHaidar, Rabih Talhouk

**Affiliations:** ^1^Department of Biology, Faculty of Arts and Sciences, American University of Beirut, Beirut, Lebanon; ^2^Department of Cell and Systems Biology, Faculty of Arts and Sciences, University of Toronto, Toronto, ON, Canada

**Keywords:** gap junctions, connexins, breast cancer, microRNAs, circularRNAs, tumor-suppressors, biomarkers, prevention

## Abstract

Breast cancer (BC) is a global public health burden, constituting the highest cancer incidence in women worldwide. Connexin43 (Cx43) is a member of a family of transmembrane proteins responsible in part for intercellular communication between adjacent breast epithelial cells, via gap junctions. Cx43 plays key role in mammary gland development and differentiation and its spatio-temporal perturbation contributes to tumorigenesis. Thus, Cx43 acts as a breast tumor-suppressor. Signaling pathways and phenotypes downstream of Cx43 mRNA loss/mis-localization in breast cells have been well-studied. However, axes parallel to Cx43 loss are less understood. microRNAs (miRNAs) are small endogenous non-coding RNAs that repress translation and circularRNAs (circRNAs) are a class of endogenous RNAs that originate from RNA splicing and act as miRNA “sponges”. CircRNAs and miRNAs are dysregulated in cancers and are highly abundant and stable in the circulation. Thus, they present as attractive liquid biopsy cancer biomarkers. Here, an axis for Cx43 mRNA-circRNAs-miRNAs interactions along BC initiation (denoted by loss of breast epithelial polarity and development of hyperplastic phenotypes) is proposed to potentially serve as a signature biomarker toward BC early-onset detection and prevention.

## Introduction

BC registers the highest incidence and mortality rates in females and is the second most commonly diagnosed cancer (after lung cancer) (1). Incidence of early-onset BC in young women is alarming and has increased drastically (2–4). It is crucial to focus on non-invasive biomarkers and active players in BC early initiation processes, toward prevention and early detection (5). The mammary gland undergoes extensive remodeling during development, from prenatal to post lactation stages (6, 7). Lobules, milk ducts, connective tissues, and adipose tissues constitute the mature human female breast. Functional centers that link a lobule to its terminal duct and to the ductal system are terminal duct lobular units (TDLUs). Each lobule contains group of alveoli, responsible for milk secretion during lactation. Both ducts and alveoli are lined by luminal epithelial cells, forming ductal and lobular epithelium, respectively, which in turn are lined by discontinuous layer of myoepithelial cells and are separated by a supporting basement membrane. The latter is underlain by the stroma, an extracellular matrix (ECM) and stromal cells, including fibroblasts, adipocytes, endothelial cells, and immune cells (8–10).

**Figure 1:**
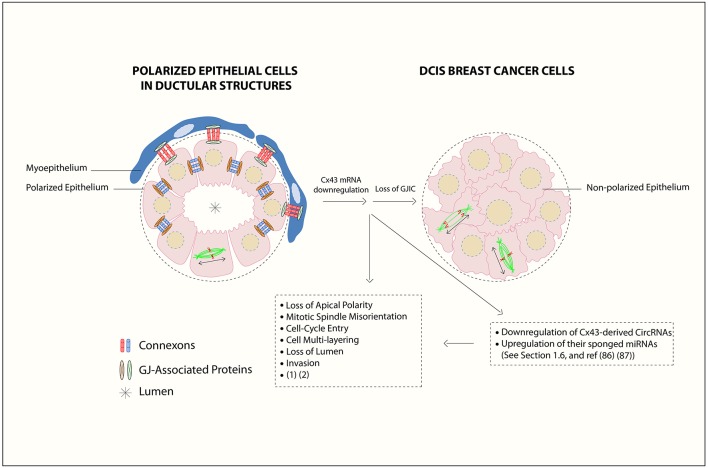
Gap junction (GJ) complex dis-assembly in breast cancer initiation. In normal differentiated mammary epithelium, the cells polarize with apical, and basolateral domains and assemble membranous GJs between epithelial cells and between epithelial and myoepithelial cells. Mammary Cxs (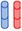), including Cx43, form a complex assembly with GJ-Associated Proteins (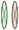) such as ZO-2, α- and β-catenins (15) in a differentiated epithelial cell. At the primary tumor site, the downregulation of Cx43 mRNA levels leads to loss of gap junction intercellular communication (GJIC) and dissociation of GJ-associated proteins complexes, which in turn causes loss of communication between neighboring cells, activation of cellular proliferation, and alteration in polarity protein distribution. Loss of apical polarity, mitotic spindle misorientation, cell cycle entry, cell multi-layering, loss of lumen (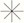), and enhanced invasive capability in Cx43 knock out breast epithelial cells is also reported (16, 17). Mitotic-spindle orientation (MSO) is depicted based on the directionality of the α-tubulin poles, either parallel to the basement membrane [or tangential to the circumference of the growing acini], which is the proper MSO to maintain a monolayered epithelium (in polarized epithelial cells in ductular structures), in contrast to cell multilayering (in DCIS breast cancer cells). Double-headed arrows indicate MSO. Thus, Cx43 contributes to breast epithelial polarity and proper MSO in single layered mammary epithelial cells, whereas its loss contributes to disrupted polarity and MSO and multilayering, which are hallmarks of tumor initiation. In this review, an axis by Cx43-derived circRNAs and their sponged miRNAs is proposed during BC initiation stages, which almost parallels the roles of Cx43 mRNA down-regulation and GJIC loss. This is denoted by loss of breast epithelial polarity and development of hyperplastic phenotypes (18, 19). The axis might act as promising biomarker signature toward BC early-onset detection and prevention, as discussed in section Cx43 mRNA-circRNAs-miRNAs Axis [Figure is modified from El-Saghir et al. (20)].

Mammary gland development requires well-orchestrated cell-cell and cell-ECM communication by gap junctions and systemic signals. Connexins (Cxs) are a family of transmembrane proteins. They are responsible for establishing gap junction intercellular communication (GJIC), capable of linking cytoplasm of two neighboring cells, allowing intercellular exchange of ions, second messengers, and metabolites (11–13). Each GJ channel is made up of two docked connexons, spanning the two membrane bilayers of adjacent cells, whereby each connexon forms by oligomerization of hexagonally arrayed connexins (14). GJs mediate channel-dependent and channel-independent functions. Any perturbations in Cxs expression/localization may alter the function of the gland and lead to tumorigenesis. Cxs act as tumor-suppressors, in a context-dependent manner, like Cx43, the focus of this review (8, 9) (Figure 1).

Recently, we revealed an apicolateral distribution/localization of Cx43 in luminal human breast epithelium, and that loss of Cx43 expression contributes to breast tumorigenesis by disrupting apical polarity and promoting cell multi-layering, a hallmark of tumor initiation (17). Furthermore, populations at higher risk of BC (like obese patients) exhibit loss of Cx43 apical distribution and cell multi-layering in an inflammatory microenvironment (21, 22). Studies from our group have characterized pathways and phenotypes downstream of Cx43 loss/mis-localization in 3D human breast epithelial HMT-3522 S1 cells (16, 17, 23–25). Hence, an axis that parallels Cx43 mRNA loss will be proposed. miRNAs are small non-coding RNAs that repress translation, and circRNAs originate from RNA splicing and act as miRNA “sponges” (26, 27). CircRNAs and miRNAs unique dysregulation signatures in cancers (in tissue- and development stage-specific manner), their tumor suppressive/oncogenic roles and stability and abundance in body fluids make them attractive non-invasive biomarkers in liquid biopsies (5, 27). Here, an axis by Cx43-derived circRNAs and their sponged miRNAs is proposed during BC initiation stages, which might act as promising biomarker signature toward BC early-onset detection and prevention, especially in patients at increased risk.

## Cx43 in Normal Mammary Gland Development and Differentiation

GJs play major role in establishing communication between adjacent cells (20, 28–30) and studying mice made it possible to infer Cxs spatio-temporal expression patterns across mammary gland development (31). The mammary gland expresses Cx43 in myoepithelial and epithelial cells junction (23), whereby Cx43 mRNA levels drop half-way through gestation and lactation, while its active phosphorylated form is evident during lactation (9). Autosomal dominant Cx43 mutant mice (Cx43^I130T/+^) exhibited delay in ductal elongation and atrophied glands pre-puberty (32). Myoepithelial contractility was inhibited upon Cx43 knockdown or GJIC blockage in primary mammary organoids of wild-type mice (33). Substituting Cx43 levels with Cx32 retarded growth and survival of (Cx43^Cx32/+^) heterozygous knock-in pups, due to perturbation in milk ejection (34). These studies confirm Cx43 pivotal role along mammary gland development. We also demonstrated crucial roles for Cx43 in mammary epithelial differentiation, which relied on proper GJ complex assembly composed of Cx43, α-catenin, β-catenin, and ZO-2 (15). Thus, studying Cx43 perturbation is important in understanding early events in breast cellular transformation.

## Perturbations in Cx43: Cx43 as Tumor Suppressor/Biomarker in BC

Since the mammary gland development is sensitive to perturbations in Cx43 expression, localization and function, Cx43 plays a tumor-suppressive role and contributes to breast tumorigenesis, in a context- and stage-dependent manner (35–39). Overexpression of Cx43 in MCF-7 and MDA-MB- 231 BC cells significantly decreased cells proliferation and nuclear levels of β-catenin in 3D cultures, which was mediated by membranous Cx43 recruitment of α-catenin, β-catenin and ZO-2 (24). McLachlan et al. (40) linked an impedance of tumor growth to upregulation of Cx43 *in vivo*, by favoring a mesenchymal to epithelial transition. Recently, we showed for the first time an apicolateral distribution and localization of Cx43 in luminal breast epithelium. Further, we showed that silencing Cx43 expression contributes to breast tumorigenesis by enhancing proliferation and cell cycle progression and inducing mis-localization of membranous β-catenin, resulting in loss of apical polarity, misorientation of mitotic spindle, cell multi-layering, and loss of lumen (hallmarks of tumor initiation). Silencing Cx43 activates signaling pathways that promote invasion in non-tumorigenic breast epithelium (16, 17). Similarly, Lesko et al. (41) showed that disruption of epithelial polarity was a marker of epithelial-derived tumor initiation.

Teleki et al. (42, 43) conducted a meta-analysis on Cx isotype expression data in breast tissue microarray from patients from all tumor grades. Their results showed, both in normal and breast tissues, the expression of Cx43, Cx46, Cx26, Cx30, and Cx32. Of the detected Cxs, only Cx43 correlated with improved disease prognosis and served as better prognostic marker than vascular invasion or necrosis. High levels of Cx43 in grade 2 tumors marked them as good relapse free survival subgroups. Other microarray results from tissue samples of invasive breast carcinoma patients showed that Cx43 levels positively correlated with progesterone and estrogen receptor status, but negatively correlated with Ki67 (proliferation marker) expression (44). In contrast, high levels of Cx43 was detected in BC patient biopsies at later tumor stages, suggesting its potential role in inducing tumor progression (45, 46). This is since during invasion, the tumor epithelial cells may reactivate GJIC with endothelial cells to facilitate intravasation/extravasation (20). Thus, Cx43 acts as a tumor suppressor in normal breast tissues, its loss/mis-localization contributes to BC initiation, its high levels in the primary tumor serves as a good prognostic marker while its re-expression at later tumor-stages facilitates invasion and metastasis (20).

## Interactions Between Connexins and microRNAs

Recent studies reported two possible modes of interaction/regulation between miRNAs and Cxs. The first through direct binding of miRNAs to 3'-UTR of mRNAs coding for Cxs and other junctional proteins, and the second via direct transfer of candidate miRNAs through gap junctions between neighboring cells. Lin et al. (47) correlated BC distant metastasis to opposite expression levels of miR-206 and Cx43 in triple-negative MDA-MB-231 cells via miR-206 direct binding to Cx43-3'UTR. Inhibition of miR-206 caused an increase in Cx43 levels with significant upregulation in cell proliferation, migration, and invasion. Chang et al. (48) showed that low expression levels of miR-30a increased BC invasion and metastasis, while rescuing miR-30a levels caused cancer cells to switch from mesenchymal to epithelial etiology, by inhibiting interactions between Slug and claudin promoter (tight junction proteins). Oligonucleotides (size of siRNAs) passed only through Cx43/Cx43 GJ channels (49) and transfer of miR-5096 between tumor and endothelial cells was mediated by GJs in co-cultures of glioblastoma (U87) and microvascular endothelial (HMEC) cells (50).

Cxs-miRNAs interactions are important not only for their regulatory roles, but also for their biomarker potential. Current available BC prognostic and diagnostic tests exhibit limitations (26). Serum antigens like carcinoembryonic antigen (CEA) and cancer antigen 153 (CA153) exhibit low sensitivity (51). Other tests require patient tissue biopsies, like Oncotype DX test, which estimates recurrence likelihood, MammaPrint, a prognostic test, and Veridex 76-gene signature, a diagnostic test that predicts distant metastasis in ER+ patients (52). Furthermore, mammograms usually display high false positive rates and do not detect cancers in young patients (53, 54). Amongst the BC diagnostic miRNAs, onco-miR-21 was significantly upregulated in plasma/serum and in frozen/ Formalin-Fixed, Paraffin-Embedded BC tissues compared to their normal counterparts in various ethnic cohorts (55). miR-155 and miR-18a were upregulated in sera and tissues of different ethnic cohorts and in sera of ER+ BC patients, respectively (26). Among the prognostic biomarkers, miR-106b predicted risk of high recurrence and shorter overall survival, while miR-122 was over-expressed in sera of relapsed patients and predicted metastasis (56). miR-18b, miR-103, miR-107, and miR-652 predicted recurrence and decreased overall survival in triple-negative BC patients (57). Therefore, Cxs and miRNAs serve as promising biomarkers for BC initiation and progression.

## CIRCULArRNAs Biogenesis, Functions, and Biomarker Roles in BC

CircRNAs are known to regulate miRNAs function and biogenesis and dysregulated mRNA-circRNAs-miRNAs axes may act as signatures in cancers (58–61). CircRNAs are generated from RNA splicing (conserved sequences AG GT) by back ligation. CircRNAs are covalently closed continuous loops without 5′ cap or 3′ polyadenylated tail and are resistant to exonucleases (e.g., RNase R), which degrade linear RNA. They are structurally stable and their isolation and purification is easy. CircRNAs are expressed in tissue- and- developmental stage-specific manner and primarily localize to the cytoplasm and function as miRNA sponges (sequestering miRNAs and enhancing mRNAs stability and translation) (62–64). Known functions of circRNAs are sponging miRNAs and RNA-binding proteins (RBP)s, regulating cell cycle (e.g., FOXO3 circRNA in BC) (65), translation of few exonic circRNAs with an open reading frame (66), acting as scaffolds in protein complexes assembly (66), protein sequestration from subcellular localization (67), modulating parental gene expression (68), and regulating alternative splicing (69, 70). CircRNAs are primarily located in the cytoplasm and are up to 10 times more abundant than their linear counterparts (71), are released from cell lines via exosomes and microvesicles (72), are differentially expressed in exosomes from mice with tumors compared to healthy controls (59) and hundreds of circRNAs are significantly upregulated in human blood compared to their linear counterparts (73).

Several studies have reported a role for circRNAs in the initiation and progression of BC through acting as competing endogenous miRNA sponges. Xie et al. (74) identified differentially expressed circRNAs in BC tissues, and described circ_0004771/miR-653/ZEB2 as potential regulatory feedback axis for treatment of BC. Knockdown of hsa_circ_0004771 and ZEB2 exhibited similar functions as using miR-653 mimics to promote growth inhibition and apoptosis in BC cells. Tang et al. (75) revealed that hsa_circ_0001982 was significantly overexpressed in tissues and cell lines, whereby circ_0001982 knockdown suppressed BC cell proliferation and invasion and induced apoptosis by targeting miR-143. Xu et al. (76) detected circTADA2A-E5 and circTADA2A-E6, among five most differentially expressed circRNAs in large cohort of triple-negative BC (TNBC) patients, whose downregulation associated with poor survival. Through sponging miR-203a-3p, and therefore restoring the expression of its target *SOCS3*, circTADA2A-E6 suppressed proliferation, migration, and invasion *in vitro* and possessed tumor-suppressive capability. Thus, circTADA2A-E6/miR-203a-3p/*SOCS3* might act as a promising prognostic biomarker in TNBC.

In a validation BC patient cohort, circ_103110, circ_104689, and circ_104821 levels were elevated and were predicted to sponge oncogenic miR-339-5p, miR-143-5p, miR-409-3p, miR-153-3p, and miR-145-5p. Moreover, circ_006054, circ_100219, and circ_406697 were downregulated and were predicted to sponge miR-298, miR-485-3p, and miR-100 (miRNAs involved in pathways in BC). Thus, these circRNAs are important promoters of carcinogenesis and may be useful biomarkers for BC (77). Nair et al. (78) identified 256, 288, and 411 tumor-specific circRNAs in triple negative, estrogen receptor positive, and HER2-positive BC subtypes, respectively, from 885 samples from The Cancer Genome Atlas. The tumor suppressor, circ-Foxo3, significantly downregulated in BC patients and cell lines (79), likely contributes to BC progression (71) and its levels significantly increase when cancer cells undergo apoptosis. Upon knockdown of endogenous circ-Foxo3, cell viability was enhanced, while its ectopic expression inhibited xenografts tumor growth and prompted stress-induced apoptosis by upregulating PUMA and downregulating p53 (79). Moreover, circ-ABCB10 was upregulated in BC and its knockdown *in vitro* suppressed proliferation and enhanced apoptosis through sponging miR-1271 (80, 81). The upregulation of circ-Amotl1 in cancer patients and cell lines exhibited tumorigenic capacity through interacting with proto-oncogene, c-myc (82).

Although there exists a correlation between obesity and loss of Cx43 apical distribution and cell multi-layering in breast epithelial tissues in an inflammatory micro-environment (21, 22), no studies have linked so far the involvement of adipocytes in regulating Cx43-derived circRNAs or their sponged miRNAs. However, few studies have reported the exchange of circRNAs between adipocytes and tumor cells in other cancers (83, 84). Through activating PRDM16 and suppressing miR-133, exosomes from gastric cancer cells shuttle ciRS-133 into pre-adipocytes, thus stimulating differentiation into brown-like cells (83). CircRNAs in exosomes secreted from adipocytes stimulated growth of hepatocellular carcinoma and decreased DNA damage by suppressing miR-34a and activating USP7/Cyclin A2 signaling pathway (84). CircRNAs thus serve as an attractive new class of cancer biomarker axes (85).

## Cx43 mRNA-circRNAs-miRNAs Axis

Cx43 acts as a tumor suppressor, its loss/mis-localization is an important player in breast tumor initiation (16), plays role in BC progression (17) and places some individuals (obese women) at increased risk of BC (21, 22). Follow-up on differential expression levels of Cx43 mRNA in breast tissues requires tissue biopsies. We thus predict that circulating Cx43-derived circRNAs and their sponged miRNAs could be indicative of Cx43 mRNA levels in tissues (86), and might serve as non-invasive biomarker signatures for breast cancer initiation and prevention.

To predict human circRNA isoforms that originate from linear Cx43 (GJA1) transcript, CircularRNA Interactome was used and three Cx43-derived circRNA isoforms (circ_0077753, circ_0077754, and circ_0077755) along with their sponged miRNAs were identified (66) (Supplementary Table 1). We propose that a drop in circulating Cx43-derived circRNAs levels might reflect downregulation of Cx43 expression in breast epithelial tissue. Most of the sponged miRNAs by all three Cx43-derived circRNAs isoforms are involved in cancer-related signaling pathways, as predicted by miRSystem database (87). These circRNAs associate with early events of breast tumorigenesis and are referred to hereafter as “initiation circRNAs.” Thus, when Cx43-derived circRNAs levels drop, their sponged miRNAs are expected to be relieved, and might be free to induce downstream cancer-initiating pathways. Indeed, upregulation of predicted sponged miRNAs by the three “initiation circRNAs” is involved in oncogenic initiation pathways, cellular multi-layering, and loss in organization in BC (18, 19). For instance, of the predicted sponged miRNAs, miR-182, miR-375, and miR-203 were found up-regulated during lobular neoplasia progression and miR-375 associated with loss of breast cellular organization and development of hyperplastic phenotypes. These miRNAs were indicative of a transition from lobular carcinoma *in situ* (LCIS), a benign precursor lesion, to invasive breast lobular carcinoma (ILC) (18, 19). Overexpression of oncomiRs, miR-21, miR-155, miR-10b, miR-373, and miR-520 was observed in many breast tumors (19), of which oncomiRs, miR-520g, and miR-520h are potentially sponged by two “initiation circRNAs.” Therefore, the axis parallel to Cx43 mRNA loss, denoted by “initiation” Cx43-derived circRNAs and their sponged miRNAs seems to recapitulate phenotypes along BC initiation.

## Conclusion

**Figure 2:**
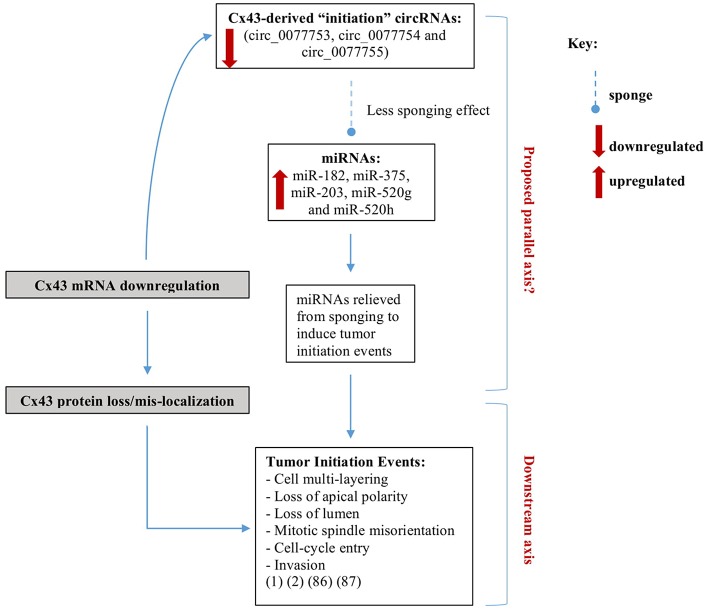
Axes parallel to and downstream of Cx43 loss in breast cancer initiation. We recently showed that silencing Cx43 expression contributes to breast tumorigenesis by enhancing proliferation and cell cycle progression and inducing mis-localization of membranous β-catenin, resulting in loss of apical polarity, misorientation of mitotic spindle, cell multi-layering, and loss of lumen (hallmarks of tumor initiation) and by activating signaling pathways that promote invasion in non-tumorigenic breast epithelium (16, 17). We propose a possible parallel signature axis of Cx43 mRNA-circRNAs-miRNAs in BC early-onset for detection and prevention, which recapitulates the roles Cx43 loss plays along breast tumorigenesis. The Cx43 mRNA- “initiation circRNAs”-miRNAs axis is denoted by three “initiation circRNAs” (circ_0077753, circ_0077754, and circ_0077755) (66) and a panel of their sponged miRNAs, miR-182, miR-375, miR-203, miR-520g, and miR-520h. When the initiation Cx43-derived circRNAs levels drop, their sponged miRNAs are expected to be relieved, and might be free to induce downstream tumor-initiation pathways (18, 19).

In this review, we propose a possible biomarker signature axis of Cx43 mRNA-circRNAs-miRNAs in BC early-onset detection and prevention. We highlighted potential regulatory roles that Cx43-derived circulating circRNAs and their sponged miRNAs may play, which almost parallels the differential roles Cx43 plays along breast tumorigenesis. The Cx43 mRNA- “initiation circRNAs”-miRNAs axis is denoted by three “initiation circRNAs” and a panel of their sponged miRNAs (identified to date in the literature), miR-182, miR-375, miR-203, miR-520g, and miR-520h. This axis, when dysregulated in breast tissues, recapitulates phenotypes due to loss of Cx43 mRNA, associated with loss epithelial polarity and cell-multilayering during initiation stages of tumorigenesis (Figure 2) (16–19).

However, circRNAs and miRNAs present with few caveats that should be addressed. Interestingly, the proposed Cx43-derived circRNAs may circumvent them. First, miRNAs and circRNAs are highly expressed in circulating blood cells and their increased levels in blood might be due to high number of blood cells. Future studies thus should focus on defining actual abundance of circRNAs in different sub-populations of blood cells, characterize their mode of transportation in serum and plasma and devise markers that predict their origin (88). Cx43, however, is abundant in endothelial cells of large arteries (at aortic and coronary arteries branch points) but not in circulating blood cells (89). Thus, Cx43-derived circRNAs in plasma and sera are expected to surpass this caveat. Secondly, some circRNAs are differentially expressed in cancer tissues compared to normal adjacent tissues, but not in plasma or sera of patients compared to healthy controls (27). Thus, Cx43-derived circRNAs can overcome this caveat through future studies that compare Cx43-derived circRNAs levels in plasma to Cx43 mRNA levels in tissues of patients at risk, patients with early-stages of the disease and those with more aggressive etiologies. Therefore, it is worth further investigating the proposed “initiation” Cx43-derived circRNAs and their sponged miRNAs signatures toward BC early-onset detection and prevention.

## Author Contributions

NN and RT assembled the relevant literature and proposed the axes. NN performed the *in silico* analysis. RT and MA mentored NN throughout the writing process and critically revised all the drafts and approved the final version for submission.

### Conflict of Interest Statement

The authors declare that the research was conducted in the absence of any commercial or financial relationships that could be construed as a potential conflict of interest.
