# Supplementary figures and images for: Connexin43 as a Tumor Suppressor: Proposed Connexin43 mRNA-circularRNAs-microRNAs Axis Towards Prevention and Early Detection in Breast Cancer
Source: Front Med (Lausanne). 2019 Aug 28;6:192. doi: 10.3389/fmed.2019.00192 (PMC6724403; doi:10.3389/fmed.2019.00192)

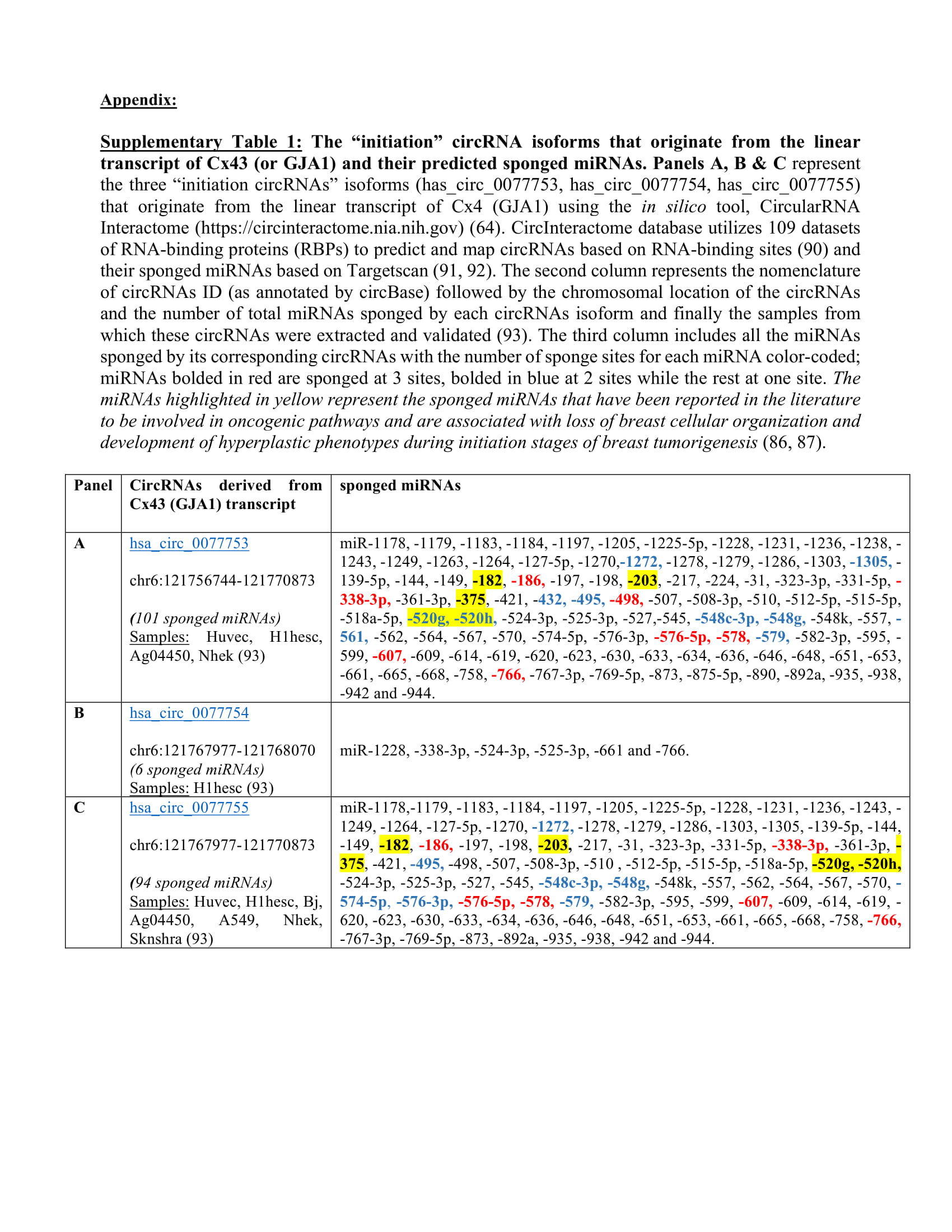

Supplement: Supplementary file 1 [file Image_1.JPEG]
